# Estimating the Global Prevalence of Zinc Deficiency: Results Based on Zinc Availability in National Food Supplies and the Prevalence of Stunting

**DOI:** 10.1371/journal.pone.0050568

**Published:** 2012-11-29

**Authors:** K. Ryan Wessells, Kenneth H. Brown

**Affiliations:** Department of Nutrition, University of California Davis, Davis, California, United States of America; Aga Khan University, Pakistan

## Abstract

**Background:**

Adequate zinc nutrition is essential for adequate growth, immunocompetence and neurobehavioral development, but limited information on population zinc status hinders the expansion of interventions to control zinc deficiency. The present analyses were conducted to: (1) estimate the country-specific prevalence of inadequate zinc intake; and (2) investigate relationships between country-specific estimated prevalence of dietary zinc inadequacy and dietary patterns and stunting prevalence.

**Methodology and Principal Findings:**

National food balance sheet data were obtained from the Food and Agriculture Organization of the United Nations. Country-specific estimated prevalence of inadequate zinc intake were calculated based on the estimated absorbable zinc content of the national food supply, International Zinc Nutrition Consultative Group estimated physiological requirements for absorbed zinc, and demographic data obtained from United Nations estimates. Stunting data were obtained from a recent systematic analysis based on World Health Organization growth standards. An estimated 17.3% of the world’s population is at risk of inadequate zinc intake. Country-specific estimated prevalence of inadequate zinc intake was negatively correlated with the total energy and zinc contents of the national food supply and the percent of zinc obtained from animal source foods, and positively correlated with the phytate: zinc molar ratio of the food supply. The estimated prevalence of inadequate zinc intake was correlated with the prevalence of stunting (low height-for-age) in children under five years of age (r = 0.48, P<0.001).

**Conclusions and Significance:**

These results, which indicate that inadequate dietary zinc intake may be fairly common, particularly in Sub-Saharan Africa and South Asia, allow inter-country comparisons regarding the relative likelihood of zinc deficiency as a public health problem. Data from these analyses should be used to determine the need for direct biochemical and dietary assessments of population zinc status, as part of nationally representative nutritional surveys targeting countries estimated to be at high risk.

## Introduction

Adequate zinc nutrition is necessary for normal pregnancy outcome and child growth, immune function and neurobehavioral development [1]. In populations at risk of zinc deficiency, preventive zinc supplementation reduces the incidence of premature delivery, decreases morbidity from childhood diarrhea and acute lower respiratory infections, lowers all-cause mortality, and increases linear growth and weight gain among infants and young children [2], [3]. In addition, therapeutic zinc supplementation during diarrheal episodes reduces the duration and severity of the illness [4].

To estimate the global and regional disease burden attributable to zinc deficiency and assess the need for and appropriate targeting of zinc intervention programs, it is necessary to determine the prevalence and severity of zinc deficiency in populations. Three indicators of population risk of zinc deficiency have been recommended: (1) the percentage of the population with plasma (serum) zinc concentrations below an appropriate cut-off, (2) the prevalence of usual dietary zinc intakes below the Estimated Average Requirement (EAR), and (3) the percentage of children less than five years of age with height-for-age Z scores less than -2 SD with respect to the WHO child growth standards [5]–[9].

Unfortunately, due to perceived high costs and logistical challenges, as well as the existence of a limited number of valid biomarkers, few nationally representative surveys have been conducted in low-income countries to assess population zinc status and the risk of zinc deficiency using the aforementioned recommended indicators. Until such data become more widely available, information on the amount of total and absorbable zinc in national food supplies may provide useful information on the risk of inadequate zinc intake in populations and help determine the need for more specific assessments of population zinc status. In a companion article to this publication, we estimated country- and region-specific risks of dietary zinc inadequacy based on national food balance sheet data obtained from the Food and Agriculture Organization (FAO) of the United Nations. The former paper highlighted the major sources of uncertainty in this analysis and evaluated the effects of different assumptions on the estimated risk of inadequate zinc intake. The present analysis focuses on the authors’ previously reported best estimates of country- and region-specific risks of dietary zinc inadequacy, generated by comparing the estimated quantities of absorbable zinc in national food supplies with the respective population’s theoretical physiological requirements for zinc. This analysis uses a newly created composite nutrient composition database, estimated physiological requirements for absorbed zinc as proposed by the International Zinc Nutrition Consultative Group (IZiNCG), a mathematical model (the Miller equation) to predict zinc absorption based on total dietary zinc and phytate and an assumed 25% inter-individual coefficient of variation in zinc intake (Wessells *et al*.).

**Figure 1:**
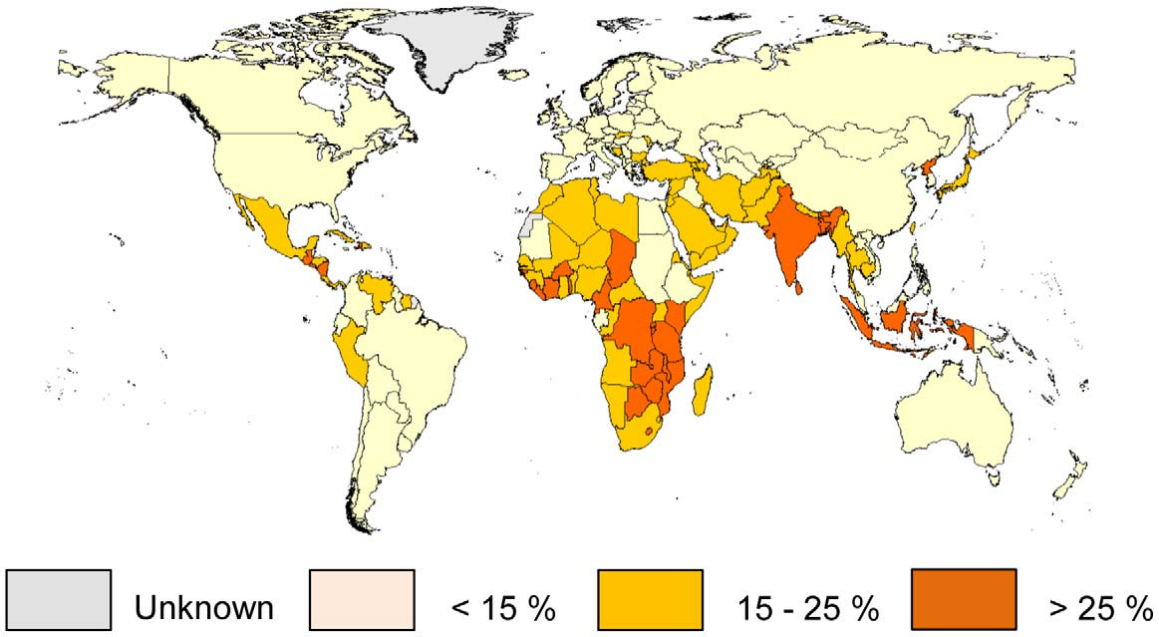
Estimated country-specific prevalence of inadequate zinc intake. Data are based on the composite nutrient composition database, IZINCG physiological requirements, the Miller Equation to estimate zinc absorption and an assumed 25% inter-individual variation in zinc intake. Data are for the 2005 time frame (2003–2007).

**Table 1:** Regional means (± SD) for national data on daily per capita energy, zinc, phytate and absorbable zinc contents of the national food supply and estimated prevalence of inadequate zinc intake in the population, years 2003–2007.

VARIABLE	High-income	Southern and Tropical L. America	China	Central and EasternEurope	Central and Andean L. America and Carib.	Central Asia, North Africa and MiddleEast	East and Southeast Asia and Pacific	Sub-Saharan Africa	South Asia	Global
**Number of Countries**	30	5	3	20	27	28	21	48	6	188
**Population (millions)**	937.2	249.4	1337.7	330.1	301.4	481.4	606.8	757.8	1495.6	6497.5
**Energy (kcal)**	3424±323	3031±88	2905±65	3286±175	2836±468	3089±359	2585±170	2351±345	2281±31	2776±472
**% of energy from ASF**	27.6±5.1	23.2±2.8	21.0±1.2	23.1±2.9	16.3±3.8	11.9±4.3	10.1±4.2	6.8±5.0	9.0±4.0	15.8±8.4
**Zinc (mg)**	13.2±1.3	12.3±0.9	13.6±0.5	11.7±1.1	10.7±3.1	13.9±3	8.9±1.3	8.4±1.7	9.8±0.7	11.4±2.5
**Zn density (mg/1000 kcal)**	3.9±0.3	4.1±0.3	4.7±0.1	3.6±0.3	3.7±0.6	4.5±0.8	3.4±0.4	3.6±0.5	4.3±0.3	4.1±0.6
**% of Zn from ASF**	59.7±8.5	59.7±7.2	49.2±2.3	51.8±4.2	39.7±9.7	23.7±11.1	28.0±10.7	19.1±11.6	11.5±4.9	34.8±20.0
**Phytate (mg)**	1173±173	1170±245	1456±18	1199±210	1887±929	2776±1089	1440±165	1782±499	2259±226	1730±649
**Phytate : zinc molar ratio**	9.0±2.2	9.6±2.3	10.6±0.8	10.1±1.3	16.6±4.6	19.1±4.5	16.4±3.1	21.3±4.4	22.8±1.1	15.8±6.2
**Est. fractional absorption**	0.25±0.01	0.26±0.01	0.23±0.00	0.27±0.02	0.26±0.07	0.19±0.05	0.28±0.02	0.27±0.04	0.23±0.02	0.24±0.04
**Absorbable zinc (mg)**	3.32±0.28	3.21±0.32	3.17±0.09	3.10±0.13	2.53±0.21	2.56±0.24	2.46±0.26	2.17±0.28	2.19±0.05	2.71±0.51
**% mean physiological requirement**	160.8±14.7	164.3±15.5	155.4±4.7	149.2±6.7	133.0±9.7	133.4±12.2	127.3±14.6	123.0±14.5	115.7±4.1	138.0±20.8
**Estimated % of pop. with inadequate zinc intake**	7.5±4.1	6.4±1.8	7.8±2.1	9.6±2.4	17.0±5.9	17.1±5.4	22.1±10.0	25.6±12.2	29.6±3.6	17.3±11.1

Estimates were calculated using the composite nutrient composition database, IZiNCG physiological requirements, the Miller Equation to estimate zinc absorption and an assumed 25% inter-individual variation in zinc intake. Regional data are presented first for high-income countries, and then in ascending order (from left to right) according to the estimated of inadequate zinc intake. Data are weighted by national population sizes and are for 188 countries. Regional classifications are based on the reporting regions of the Global Burden of Diseases, Injuries, and Risk Factors 2010 Study, and are grouped according to geographical location and dietary patterns (Table S1). ASF, animal source foods.

FAO food balance sheets supply data on annual national food availability, and do not account for differences in dietary zinc intake among individuals and sub-groups within the population. Of particular concern, food balance sheets may be more likely to represent food intake by adults than by infants and young children, who are likely more vulnerable to zinc deficiency than others in the population [1], [10], [11]. Thus, food balance sheets may not provide a good estimate of inadequate zinc intake by young (pre-school aged) children. On the other hand, the prevalence of low height-for-age in children under 5 years of age in a specific population reflects pre- and post-natal nutritional conditions of young children and has been recommended as an indirect indicator of a population’s risk of zinc deficiency. When the prevalence of stunting is greater than 20%, the risk of zinc deficiency may also be elevated [9]. By using both food balance sheet information and the prevalence of stunting, it may be possible to estimate the risk of zinc deficiency in the whole population, including both older children and adults and pre-school children.

The objectives of the present study were to use the estimated country- and region-specific prevalence of dietary zinc inadequacy and country-specific rank order of estimated prevalence to: (1) examine dietary patterns associated with the estimated prevalence of inadequate zinc intake, (2) evaluate country-specific secular trends in the estimated prevalence of inadequate zinc intake, and (3) compare the estimated prevalence of dietary zinc inadequacy with the national prevalence of stunting in children less than five years of age and create a composite index to identify countries at the highest risk of zinc deficiency, based on both indicators. These analyses were conducted as part of the Nutrition Impact Model Study (NIMS), which was designed to synthesize information related to the health impacts of nutritional conditions and deficiencies and related interventions, in developing countries.

**Figure 2:**
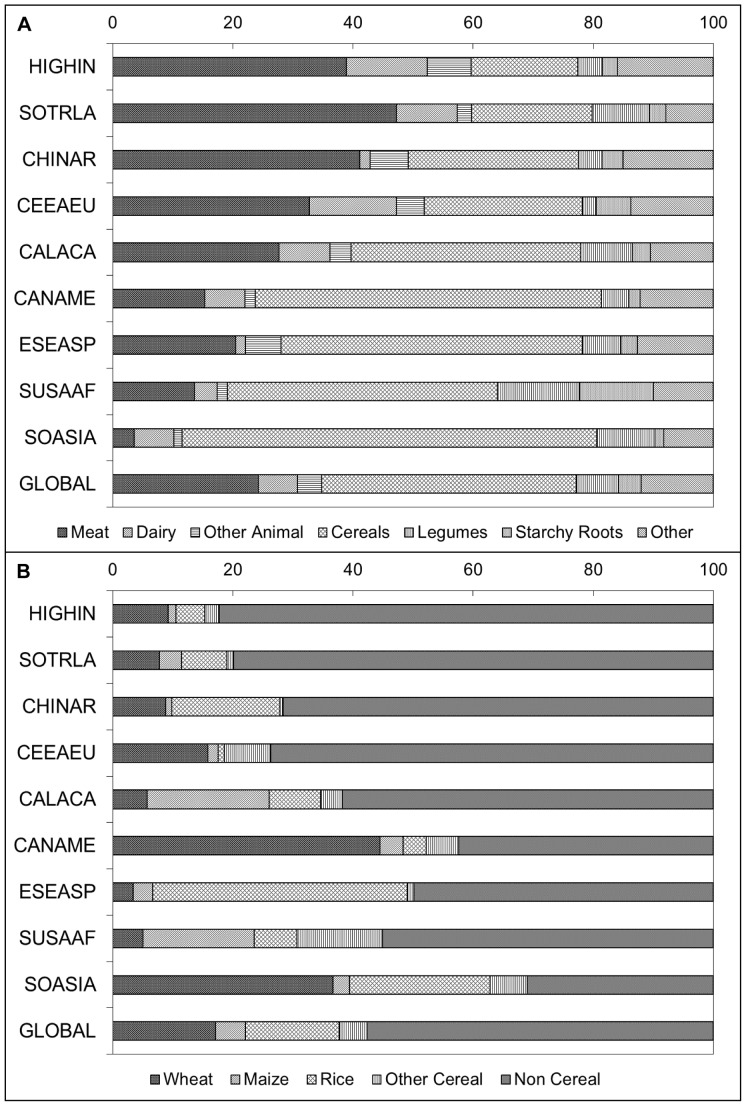
Percentage of total zinc in national food supplies derived from (a) all food sources and (b) cereal and non-cereal sources. Regional data are weighted by national population size and listed in ascending order according to the estimated prevalence of inadequate zinc intake in the region. HIGHIN, High-income; SOTRLA, Southern and Tropical Latin America; CHINAR, China; CEEAEU, Central and Eastern Europe; CALACA, Central and Andean Latin America and the Caribbean; CANAME, Central Asia, North Africa and the Middle East; ESEASP, East and South-East Asia and the Pacific; SUSAAF, Sub-Saharan Africa; SOASIA, South Asia. Data are for the 2005 time frame (2003–2007).

**Figure 3:**
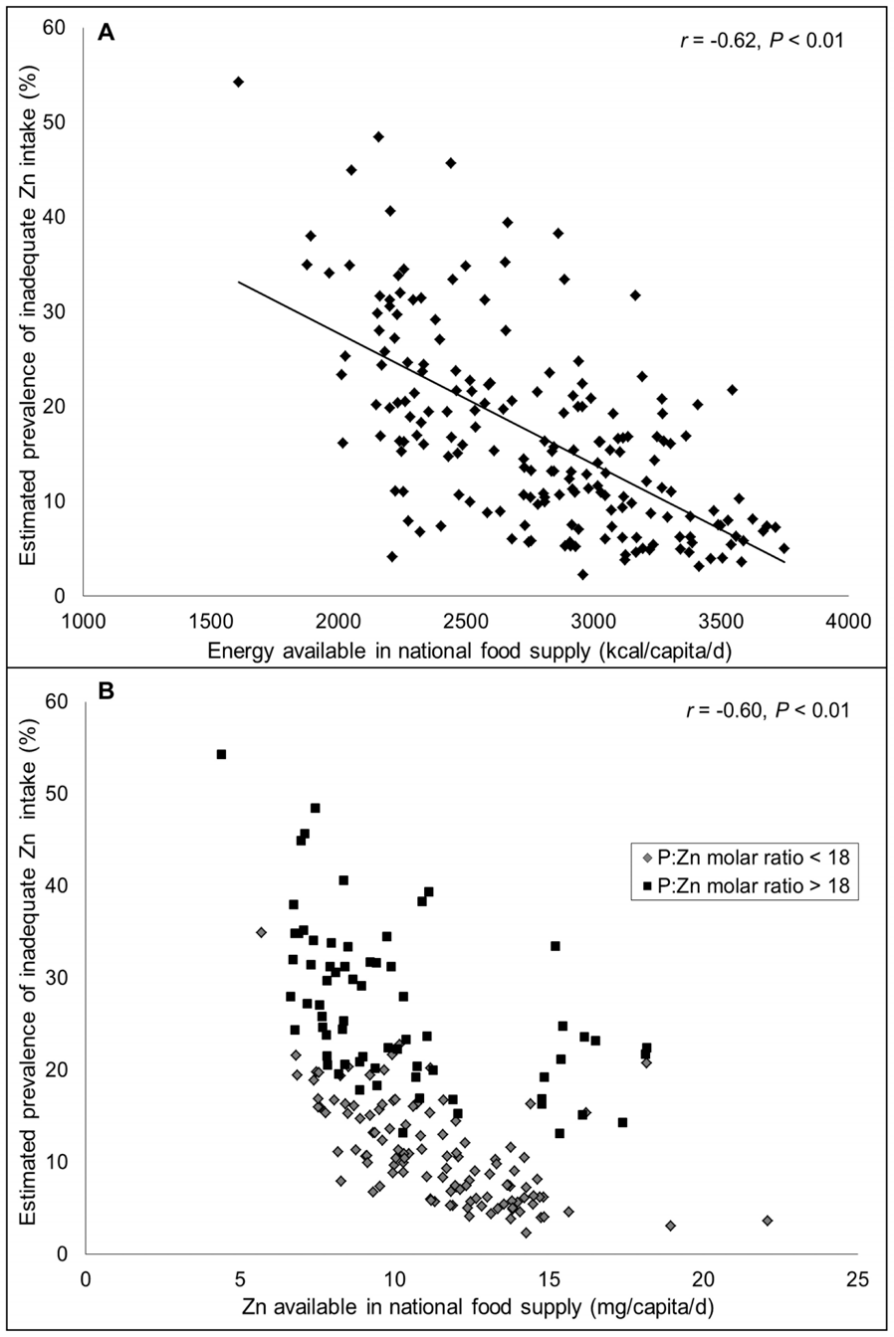
Relationship between availability of (a) energy (kcal/capita/d) and (b) total zinc (mg/capita/d) in the national food supply and the estimated prevalence of inadequate zinc intake. N = 188. Data are for the 2005 time frame (2003–2007).

**Figure 4:**
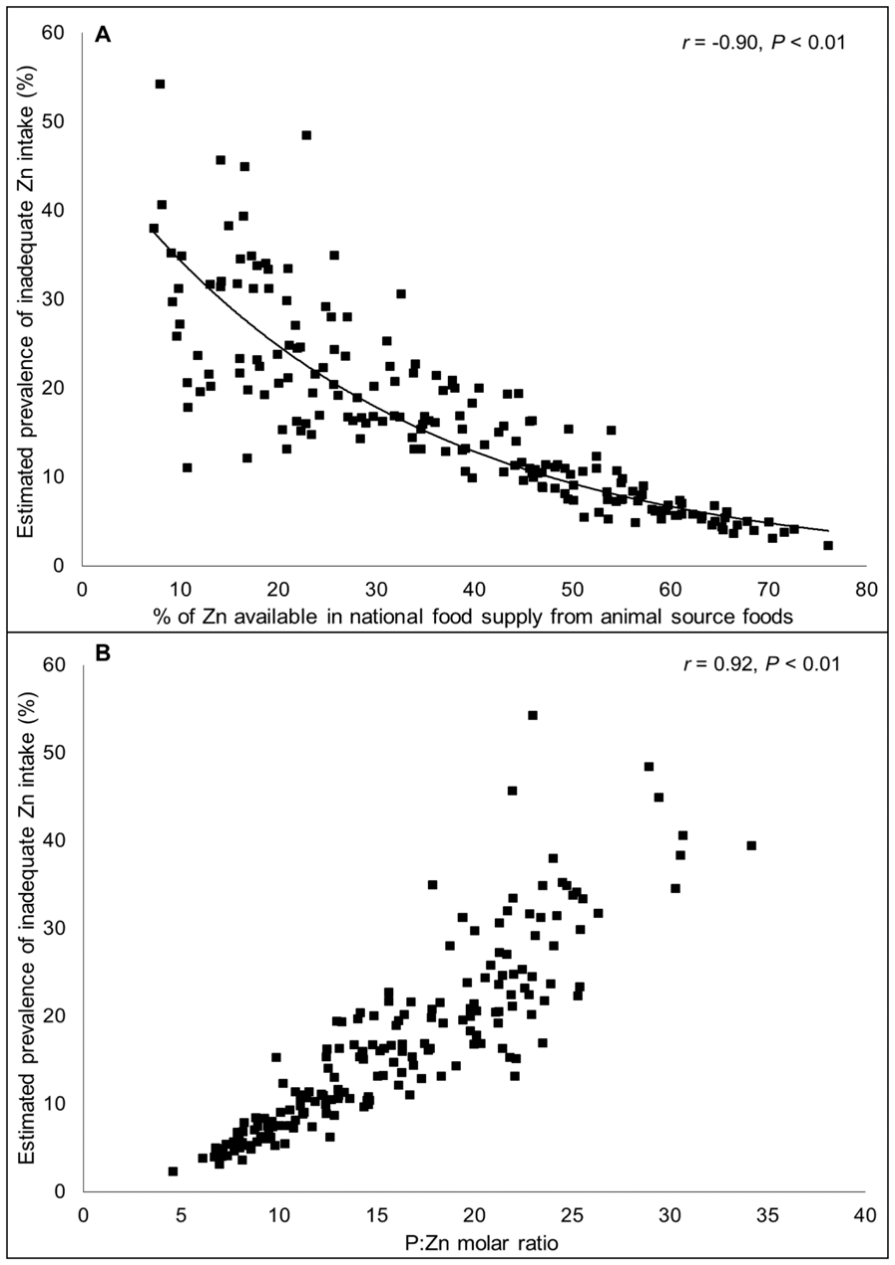
Relationship between (a) the percentage of zinc from animal source foods and (b) the phytate:zinc molar ratio in the national food supply and the estimated prevalence of inadequate zinc intake. N = 188. Data are for the 2005 time frame (2003–2007).

## Methods

### Estimation of the Adequacy of Zinc in National Food Supplies Based on National Food Balance Data

The analytic methods, and model assumptions, have been described extensively in the accompanying methodological article (Wessells *et al.*). In brief, the following steps were completed to estimate the national prevalence of inadequate zinc intake and calculate the country-specific rank order of estimated prevalence. Firstly, we obtained country-specific data on the average daily per capita availability of major food commodities (kcal/capita/d) from national food balance sheets. These data are provided by 188 countries to the Food and Agriculture Organization of the United Nations and are available in the public domain. We then calculated the zinc and phytate contents of each food commodity, using a composite nutrient composition database created for the purpose of these analyses and accounting for food processing methods. The total zinc and phytate contents of the daily food supply were calculated as the sum of the zinc and phytate contents of each commodity food. Data for the new composite database were obtained from the WorldFood System International Mini-list (IML) [12], the Nutrition Data System for Research Version 2010 (NDSR, Nutrition Coordinating Center, University of Minnesota) [13], the USDA Nutrient Database for Standard Reference, Release 23 (USDA SR23) [14], the INFOODS Regional Nutrient Database for West Africa [15], *Food Phytates,* edited by Reddy *et al* [16], and current scientific literature. Subsequently, we estimated the absorbable zinc content of the daily food supply on a per country basis, using the Miller Equation, which is a saturation response model of zinc absorption as a function of dietary zinc and phytate [17], [18]. This method allowed us to predict the fractional absorption of zinc and the absorbable zinc content of the daily food supply for each country. Next, we calculated the theoretical mean daily per capita physiological requirement for zinc, based on the age and sex distribution of the national population and using recommendations developed by IZiNCG. Population data were obtained from the Institute for Health Metrics and Evaluation (IHME, University of Washington) based on the 2010 Revision of the World Population Prospects, which is available from the Population Division of the Department of Economic and Social Affairs of the United Nations. We then calculated the percentage of the mean physiological requirement for zinc that is available in the national food supply, by dividing the estimated absorbable zinc content of the national food supply by the calculated national physiological requirement. Finally, we estimated the prevalence of inadequate zinc intake, using a method akin to the IOM EAR cut-point method and assuming a 25% inter-individual coefficient of variation (CV), and calculated country-specific rank order of estimated prevalence [19]. We designated populations as being at moderate- or high-risk of zinc deficiency when the percentage of the population at risk of inadequate zinc intake due to inadequate zinc in the food supply was 15–25% and >25% respectively.

**Figure 5:**
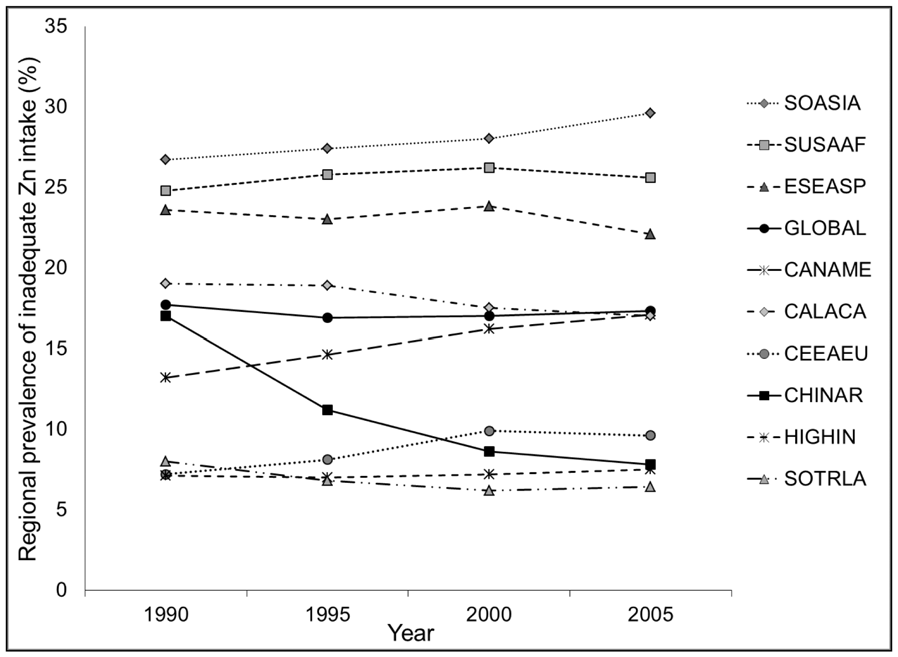
Secular trends in the global and regional estimated prevalence of inadequate zinc intake between 1990 and 2005. SOASIA, South Asia; SUSAAF, sub-Saharan Africa; ESEASP, East and South-East Asia and the Pacific; CANAME, Central Asia, North Africa and the Middle East; CALACA, Central and Andean Latin America and the Caribbean; CEEAEU, Central and Eastern Europe; CHINAR, China; HIGHIN, High-income; SOTRLA, Southern and Tropical Latin America.

To examine secular trends in the adequacy of zinc in national food supplies, and to smooth differences in inter-year variability (due to mistakes in reporting, drought, etc.), we created estimates of the percentage of the population at risk of inadequate intake over four five-year periods encompassing years of interest: 1990 (1988–1992), 1995 (1993–1997), 2000 (1998–2002) and 2005 (2003–2007).

### Stunting

Stunting data were obtained from a recent systematic analysis, which used population-representative data on height-for-age z-score (HAZ), calculated using the WHO child growth standards, to estimate the prevalence of stunting for 141 low- and middle-income countries [20]. The WHO considers stunting to be a public health problem when the prevalence of stunting among children less than 5 years of age is >20% [21]. Using the aforementioned data, we compared national estimates of the prevalence of inadequate zinc intake, based on national food balance sheet data, with the prevalence of stunting in children less than five years of age. In addition, we evaluated the relationship between secular trends in the estimated prevalence of inadequate zinc intake and the prevalence of stunting.

### Composite Index

As both the estimated prevalence of inadequate zinc intake and the prevalence of stunting provide only suggestive evidence for the risk of zinc deficiency, we created a composite index based on both indicators. Individual countries were classified into one of four categories: (1) the estimated prevalence of inadequate zinc intake is >25% and the prevalence of stunting is >20%, (2) the estimated prevalence of inadequate zinc intake is <25% and the prevalence of stunting is >20%, (3) the estimated prevalence of inadequate zinc intake is >25% and prevalence of stunting is <20%, or (4) estimated prevalence of inadequate zinc intake is <25% and prevalence of stunting is <20%.

### Statistical Analyses

Regional classifications are based on the reporting regions of the Global Burden of Diseases, Injuries, and Risk Factors 2010 Study, and are grouped according to geographical location and dietary patterns **(Table S1)** [22]; individual country data are available to re-group countries using other classification systems, such as WHO regions (**Table S2**). Regional and global data were weighted by national population sizes. Bivariate associations between the estimated prevalence of inadequate zinc intake, dietary patterns, and the prevalence of stunting were assessed with Spearman correlations. All statistical analyses were completed using SAS System for Windows release 9.3 (SAS Institute, Cary, North Carolina). Data are presented as means±SD, unless otherwise noted. A *P* value <0.05 was considered statistically significant.

**Figure 6:**
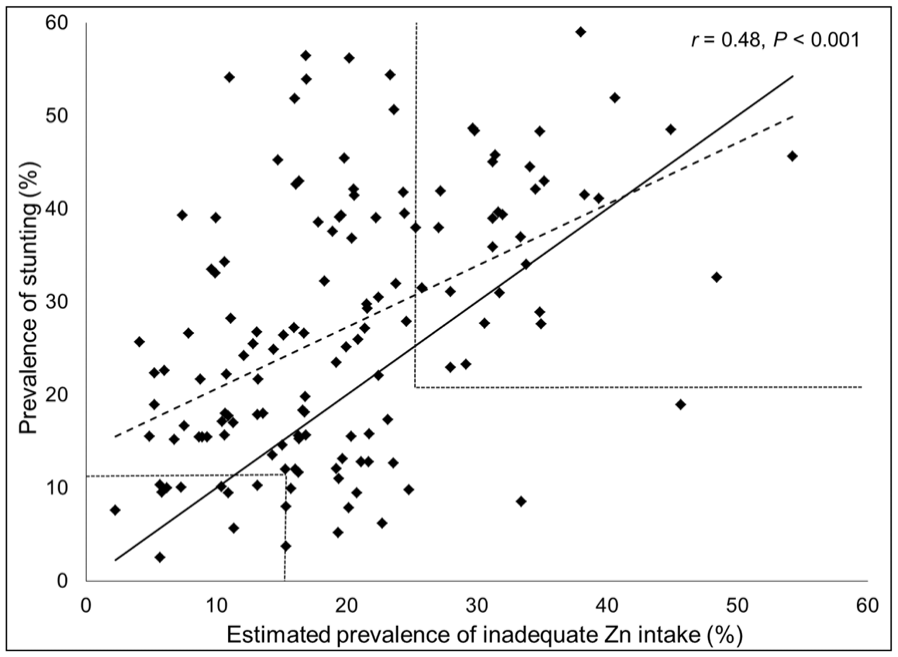
Relationship between the estimated prevalence of inadequate zinc intake and the prevalence of childhood stunting. Stunting data (low height-for-age) are for children less than five years of age in138 low- and middle-income countries. The solid line represents the line of identity (intercept = 0, slope = 1). The dashed line represents the best-fit regression line. Dotted lines demarcate prevalence data associated with low, moderate and high risk of inadequate zinc intake, based on the composite index of both variables.

**Figure 7:**
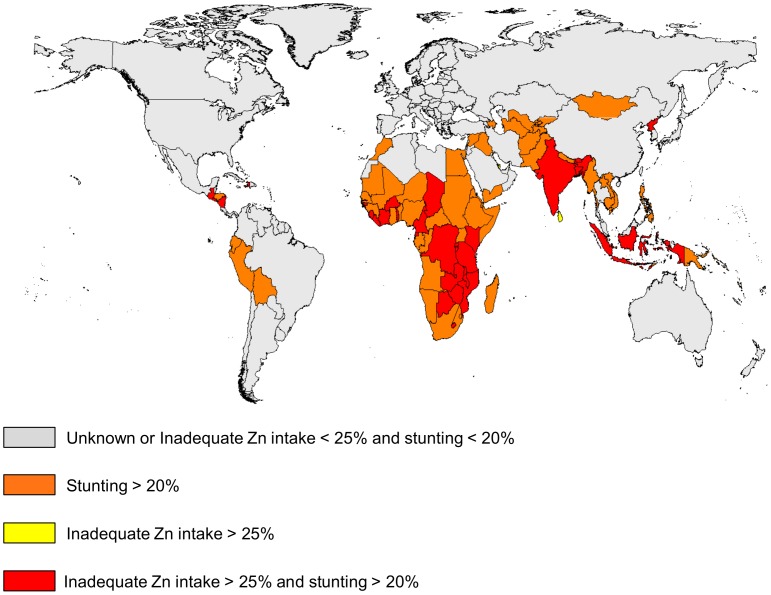
National risk of zinc deficiency based on the prevalence of childhood stunting and the estimated prevalence of inadequate zinc intake. Stunting data (low height-for-age) are for children less than five years of age in138 low- and middle-income countries. The estimated prevalence of inadequate zinc intake is based on the FAO national food balance sheet data, the composite nutrient composition database, IZINCG physiological requirements, the Miller Equation to estimate zinc absorption and an assumed 25% inter-individual variation in zinc intake.

## Results

Regional and global means (± SD), weighted by national population sizes, of the percentage of the mean physiological requirement for zinc that is available in the regional food supply and the estimated prevalence of inadequate zinc intake for the period 2003–2007 are presented in **Table 1**. Also included are data on the daily per capita energy, zinc, phytate, absorbable zinc contents of the regional food supplies and the percent of energy and zinc derived from animal source foods. Data are first presented for high-income countries, and then for other regions in ascending order according to the estimated prevalence of inadequate zinc intake. Based on this model, the global food supply provides ∼138% of the physiological requirement for absorbed zinc, weighted by national population size. An estimated 17.3% of the global population is at risk of inadequate zinc intake. The regional estimated prevalence of inadequate zinc intake ranged from 7.5% in high-income regions to 30% in South Asia. Within regions, individual countries had a fairly consistent estimated prevalence of inadequate zinc intake, with specific countries in South and South-East Asia, Sub-Saharan Africa, and Central America having the greatest risk of inadequate zinc intake **(** **Figure 1** **)**. National data for the estimated prevalence of inadequate zinc intake for 188 countries based on food balance sheet data, as well as country-specific rank order by estimated prevalence, using the 2003–2007 time frame estimates, are available as online supporting material **(Table S2).**

### Composition of National and Regional Food Supplies

The estimated proportion of total zinc in national food supplies that is derived from various food sources is depicted in **Figure 2** **,** by geographical region and weighted by national population size. Regions are listed in ascending order according to the estimated prevalence of inadequate zinc intake in the population. Total dietary zinc availability was closely associated with energy availability, as zinc densities (mg/1000 kcal) among regions were fairly constant. As the total energy and zinc contents of the food supply increased, the estimated prevalence of risk of inadequate zinc intake decreased (*r* = −0.62 and −0.60, respectively; *P*<0.01) **(** **Figure 3** **)**. The absorbable zinc content of the national food supplies was associated with the percentage of energy and zinc obtained from animal source foods and the P:Zn molar ratio, as well as total energy availability. The percent of total dietary zinc available from animal source foods in the food supply was negatively correlated with the estimated prevalence of inadequate zinc intake (*r* = −0.90, *P*<0.01) **(** **Figure 4a** **).** The mean percentages of dietary zinc obtained from animal source foods in countries identified as having at low, moderate and high estimated prevalence of inadequate zinc intake were 51.2%, 27.1% and 12.1%, respectively. Total dietary phytate and the P:Zn molar ratio were positively correlated with the risk of inadequate zinc intake (*r* = 0.62 and 0.92, respectively; *P*<0.01) **(** **Figure 4b** **).** With just one exception each, all countries with P:Zn molar ratio <12 were considered to be at low risk for inadequate zinc intake (estimated prevalence <15%) and all countries with a P:Zn molar ratio >18 were considered to be at high risk of inadequate zinc intake (estimated prevalence >25%).

### Secular Trends in the Composition of National Food Supplies

Global and regional estimates of zinc available in national food supplies were relatively stable over the four time intervals, with just a few exceptions **(** **Figure 5** **).** A secular trend is noted for China, where the estimated percentage of the population at risk of inadequate zinc intake decreased from 17% in 1990 to 8% in 2005, concomitant with marked increases in total zinc availability in the food supply and an increased percentage of dietary zinc available from animal source foods. Approximately 12% of countries experienced a >5% absolute reduction in the estimated prevalence of inadequate zinc intake between 1990 and 2005; however, the majority of these countries remained at moderate to high risk of inadequate zinc intake. Reductions in the estimated prevalence of inadequate zinc intake were characterized by increases in the amount of absorbable zinc in the national food supply per capita, primarily due to increases in the availability of dietary energy, increases in the percent of energy and Zn obtained from animal source foods, and/or reductions in mean P:Zn molar ratios **(Table S3).** An additional ∼12% of countries experienced a >5% increase in the estimated prevalence of inadequate zinc intake over the same time frame; all of these countries except one were at moderate to high risk of inadequate zinc intake in 2005 **(Table S4).** Increases in the estimated prevalence of inadequate zinc intake were characterized either by changes in the food supply in the opposite direction as described above or by an increased mean physiological requirement due to shifting population demographics.

### Composition of National Food Supplies and Stunting Prevalence

The estimated prevalence of inadequate zinc intake was significantly correlated with the prevalence of stunting (low height-for-age) in children under five years of age (*r* = 0.48, *P*<0.001); although there was considerable variability about the best-fit line **(** **Figure 6** **)**. 84 of 141 (59.6%) of low- and middle-income countries had a stunting prevalence >20%. Using the composite index of both indicators (estimated prevalence of inadequate zinc intake >25% *and* prevalence of stunting >20%), 32 of these aforementioned countries were identified as being at high-risk of inadequate zinc intake **(** **Figure 7** **)**.

### Secular Trends between Composition of National Food Supplies and Stunting Prevalence

Country-specific secular trends in the prevalence of stunting were not correlated with secular trends in the estimated prevalence of inadequate zinc intake (*r = *0.10; *P* = 0.24) **(Figure S1)**. Globally, the prevalence of stunting in low- and middle-income countries decreased from 43.0% to 30.3% between 1990 and 2005. However, this decrease was not mirrored by a concomitant reduction in the estimated prevalence of inadequate zinc intake in these same countries (20.7% to 19.6% between 1990 and 2005, respectively).

## Discussion

Information available from FAO food balance sheets indicates that the zinc content of national food supplies may be inadequate to meet zinc requirements for approximately 15–20% of the world’s population. Global estimates of the prevalence of inadequate intake were relatively stable over the 20-year time frame evaluated in this analysis, although secular trends were noted in ∼25% of countries, due to either changes in the food supply or demographic profile, and hence estimated national zinc requirements. The estimated prevalence of inadequate zinc intake varied by composition of the national food supply, notably total energy and zinc availability, the percentage of dietary zinc available from animal source foods and the P:Zn molar ratio. The estimated prevalence of inadequate zinc intake was negatively associated with the percent of dietary zinc obtained from animal source foods, which are relatively rich sources of zinc and do not contain inhibitors of zinc absorption. In contrast, the estimated prevalence of inadequate zinc intake was strongly positively associated with the P:Zn molar ratio, which affects zinc bioavailability.

The estimated prevalence of inadequate zinc intake are based on the current “best-estimate” model, comprised of zinc and phytate data from the composite nutrient database, IZiNCG physiological requirements for absorbed zinc, the Miller Equation to estimate the fractional absorption of zinc, and an assumed 25% CV in inter-individual intake. This model owes it strength to the thoroughness of the review of food composition databases, regional food processing techniques, and zinc requirements and absorption. However, as discussed in the accompanying methodological article (Wessells *et al.*), there is substantial variation in prevalence estimates when the model assumptions are modified. Thus, caution is advised in the interpretation of the absolute numeric prevalence estimates and the application of these results. Instead, country-specific rank order of the likely risk of inadequate intake, which is fairly consistent regardless of the model assumptions, should be used to draw inter-country inferences regarding relative likelihood of zinc deficiency as a public health problem. The data can be used to determine the need for more targeted assessments of population zinc status. Plasma zinc concentration and dietary zinc intake, which are the recommended biochemical and dietary indicators, respectively, of zinc status in populations should be measured as part of nationally representative nutritional status surveys. Based on zinc availability in their national food supplies, countries in South and Southeast Asia, Sub-Saharan Africa and Central America which were identified as being at highest risk of inadequate zinc intake should be prioritized for biochemical and dietary assessments of population zinc status.

Due to the national level data informing the estimates of the prevalence of inadequate zinc intake, we were required to assume that the ratio of zinc intake to zinc requirement is uniform across the population and we were not able to account for age-related differences in the distribution of food to individuals. Because the types of food consumed and the adequacy of food intakes by young children may differ substantially from those of adults in the same population, food balance sheet data may be more reflective of adult dietary intakes than intakes by children. Studies of preventive zinc supplementation have found that increasing zinc intake in at-risk populations increases children’s weight gain and linear growth, thereby reducing the prevalence of stunting [2]. Thus, a portion of stunting is attributable to inadequate zinc intake, and the prevalence of stunting among young children can be used as an indirect indicator of population zinc status [9]. In low- and middle-income countries, the mean prevalence of stunting in children less than 5 years of age from 2003–2007 was 30.3% [20]. In the present analyses, we found that the prevalence of stunting was positively correlated with the estimated prevalence of inadequate zinc intake. The mean prevalence of stunting in countries identified as being at low, moderate and high risk of inadequate zinc intake were 19.6%, 28.8% and 43.2%, respectively. However, in low- and middle-income countries, the mean prevalence of stunting was greater than the mean estimated prevalence of inadequate zinc intake (19.6%), and country-specific changes in the prevalence of stunting over time were not associated with parallel changes in the prevalence of the estimated risk of inadequate zinc intake. It is likely that the prevalence of zinc deficiency is higher in children under five years of age than in the general population, owing to higher nutrient density needs and rates of infection among infants and young children in low- and middle-income countries. As a result, we would expect the prevalence of stunting in a population to be higher than the estimated prevalence of inadequate zinc intake based on the adequacy of zinc in the national food supply. In addition, both indicators only provide suggestive evidence of zinc deficiency and the causes of childhood stunting are multi-factorial, which may provide some explanation for the considerable variability around the “best-fit” line.

The use of FAO food balance sheets to estimate the adequacy of zinc in national food supplies provides valuable suggestive evidence of the risk of inadequate zinc intake in respective populations, and thus the population risk of zinc deficiency. As the adequacy of zinc in the national food supply may be more likely to reflect the risk of zinc deficiency among adults, the inclusion of information on the prevalence of childhood stunting (more likely to be reflective of child risk of zinc deficiency) may provide a more comprehensive estimate of a population’s risk of zinc deficiency when using indirect indicators [1], [9]. Direct indicators of population zinc status, including plasma zinc concentration and dietary zinc intake, need to be assessed as part of nationally representative nutritional assessment surveys. As this information becomes available, these data can be used to further refine and validate the use of FAO food balance sheets and stunting prevalence to estimate the risk of inadequate zinc intake in populations.

## Supporting Information

Figure S1 **Relationship between the absolute change in the estimated prevalence of inadequate zinc intake and the change in the prevalence of stunting.** Stunting (low height-for-age) data are for children under five years of age in138 low- and middle-income countries between 1990 and 2005. The solid line represents the regression line.
(TIF)

Table S1 **Regional classifications.**
(DOCX)

Table S2 **National data on mean daily per capita energy, zinc, phytate and absorbable zinc contents of the national food supply, and estimated prevalence of inadequate zinc intake for 188 countries from 1990–2005.** Estimates were calculated using the composite nutrient composition database, IZiNCG physiological requirements, the Miller Equation to estimate zinc absorption and an assumed 25% inter-individual variation in zinc intake.
(XLS)

Table S3 **Percent change in per capita energy, zinc and phytate content of the national food supply, and percent of dietary zinc obtained from animal source foods (ASF) for countries with a >5% absolute reduction in the prevalence of inadequate zinc intake between 1990 and 2005.**
(DOCX)

Table S4 **Percent change in per capita energy, zinc and phytate content of the national food supply, and percent of dietary zinc obtained from animal source foods (ASF) for countries with a >5% absolute increase in the prevalence of inadequate zinc intake between 1990 and 2005.**
(DOCX)
