# Supplementary figures and images for: Estimating the Global Prevalence of Zinc Deficiency: Results Based on Zinc Availability in National Food Supplies and the Prevalence of Stunting
Source: PLoS One. 2012 Nov 29;7(11):e50568. doi: 10.1371/journal.pone.0050568 (PMC3510072; doi:10.1371/journal.pone.0050568)

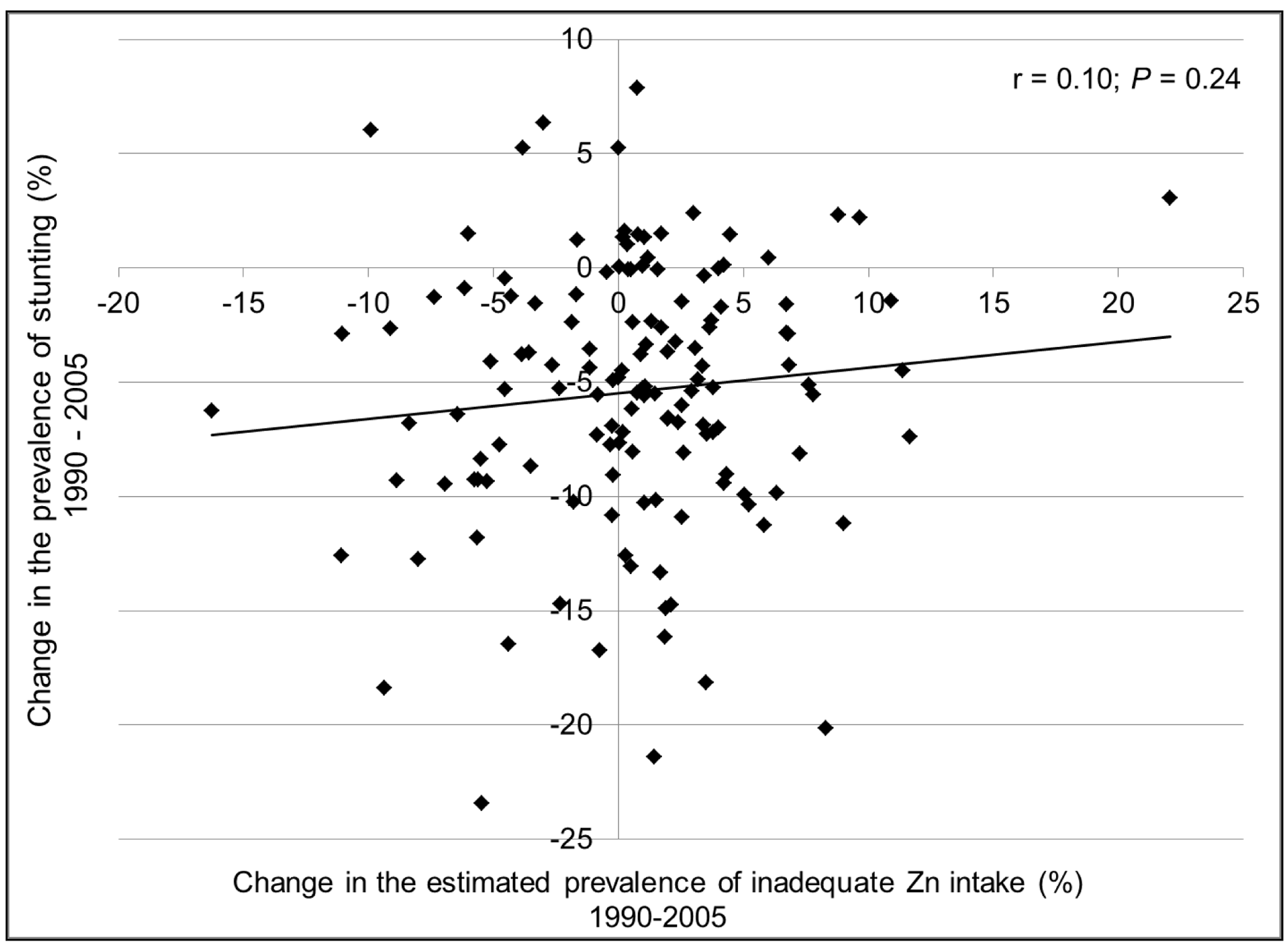

Supplement: Figure S1 — Relationship between the absolute change in the estimated prevalence of inadequate zinc intake and the change in the prevalence of stunting. Stunting (low height-for-age) data are for children under five years of age in138 low- and middle-income countries between 1990 and 2005. The solid line represents the regression line. (TIF) [file pone.0050568.s001.tif]
