# Supplementary material for: Estimating the Global Prevalence of Zinc Deficiency: Results Based on Zinc Availability in National Food Supplies and the Prevalence of Stunting
Source: PLoS One. 2012 Nov 29;7(11):e50568. doi: 10.1371/journal.pone.0050568 (PMC3510072; doi:10.1371/journal.pone.0050568)
Supplement: Table S3 — Percent change in per capita energy, zinc and phytate content of the national food supply, and percent of dietary zinc obtained from animal source foods (ASF) for countries with a >5% absolute reduction in the prevalence of inadequate zinc intake between 1990 and 2005. (DOCX) [file pone.0050568.s004.docx]

| Country | Energy (kcal) | Zinc (mg) | Zinc from ASF (%) | Phytate (mg) |
| --- | --- | --- | --- | --- |
| Albania | 11.1 | 46.9 | 31.6 | -2.4 |
| Cape Verde* | 2.7 | 0.8 | 57.2 | -24.4 |
| China | 15.3 | 44.2 | 36.0 | 0.8 |
| Congo* | 23.2 | 17.2 | 2.2 | 9.3 |
| Ecuador | 3.1 | 25.8 | 22.6 | -0.6 |
| El Salvador** | 9.6 | 15.7 | 47.5 | -2.3 |
| Guatemala** | -6.6 | -6.5 | 47.2 | -19.4 |
| Guyana | 14.4 | 40.3 | 29.7 | 21.3 |
| Haiti** | 5.9 | 0 | 38.9 | -20.0 |
| Honduras* | 12.3 | 14.0 | 37.1 | -5.0 |
| Jamaica | 9.7 | 20.8 | 8.5 | 6.7 |
| Lao PDR | 10.1 | 23.6 | 35.3 | 10.1 |
| Malawi** | 9.6 | 2.5 | -2.4 | -3.3 |
| Mali* | 15.1 | 17.4 | -3.5 | 6.2 |
| Mozambique** | 13.6 | 17.7 | 58.4 | 9.9 |
| Myanmar* | 20.6 | 39.4 | 83.6 | 22.5 |
| Nicaragua** | 24.4 | 27.1 | 25.8 | 16.1 |
| Niger* | 10.5 | 22.7 | 15.2 | 9.6 |
| Peru* | 9.3 | 32.8 | -8.4 | 28.1 |
| Sao Tome and Principe** | 14.7 | 15.0 | 42.3 | -3.0 |
| Sierra Leone** | 7.5 | 20.0 | 5.3 | 25.1 |
| Viet Nam | 28.4 | 48.1 | 57.0 | 25.2 |

*Countries at a moderate risk of inadequate zinc intake (estimated prevalence 15-25%) in 2005. **Countries at a high risk of inadequate zinc intake (estimated prevalence >25%) in 2005.
