# Supplementary material for: Estimating the Global Prevalence of Zinc Deficiency: Results Based on Zinc Availability in National Food Supplies and the Prevalence of Stunting
Source: PLoS One. 2012 Nov 29;7(11):e50568. doi: 10.1371/journal.pone.0050568 (PMC3510072; doi:10.1371/journal.pone.0050568)
Supplement: Table S4 — Percent change in per capita energy, zinc and phytate content of the national food supply, and percent of dietary zinc obtained from animal source foods (ASF) for countries with a >5% absolute increase in the prevalence of inadequate zinc intake between 1990 and 2005. (DOCX) [file pone.0050568.s005.docx]

| Country | Energy (kcal) | Zinc (mg) | Zinc from ASF (%) | Phytate (mg) |
| --- | --- | --- | --- | --- |
| Azerbaijan* | 21.3 | 20.5 | -29.0 | 30.2 |
| Bhutan** | 0.2 | -1.1 | 0.8 | -1.5 |
| Botswana** | 1.2 | -8.0 | -17.7 | -4.9 |
| Bulgaria* | -15.8 | -24.8 | -6.9 | -16.3 |
| Burundi* | -9.0 | -19.3 | -2.7 | -23.4 |
| Cuba* | 13.3 | 23.1 | -37.2 | 74.6 |
| DPR Korea** | -5.1 | -14.3 | -18.5 | -15.3 |
| DR Congo** | -27.5 | -27.9 | -1.2 | -24.8 |
| Gambia** | -5.9 | -6.8 | 2.4 | -0.8 |
| Iran (Islamic Republic of)* | 4.9 | 2.5 | 17.1 | -2.4 |
| Kuwait* | 14.4 | 5.2 | -12.9 | 14.7 |
| Latvia | -3.8 | 0.0 | -18.1 | 25.7 |
| Lesotho* | 4.3 | 2.8 | -12.9 | 5.7 |
| Liberia** | -7.6 | -12.5 | -9.9 | -6.5 |
| Libyan Arab Jamahiriya* | -2.2 | -9.1 | -18.0 | -0.2 |
| Madagascar* | -5.5 | -13.8 | -28.1 | 0.8 |
| Oman* | 1.7 | 1.3 | -0.5 | 1.0 |
| Qatar** | -1.1 | -1.9 | -2.8 | -1.2 |
| Republic of Moldova* | -2.6 | -11.0 | -19.8 | 7.2 |
| Saudi Arabia* | 9.8 | 4.9 | 2.0 | 7.1 |
| Slovakia* | -2.3 | -17.2 | -13.1 | -5.4 |
| Tajikistan* | -10.1 | -23.7 | -32.0 | -8.4 |
| Turkey* | -3.9 | -7.7 | -0.6 | -7.6 |
| United Arab Emirates* | -2.8 | -4.7 | -23.9 | 17.0 |

*Countries at a moderate risk of inadequate zinc intake (estimated prevalence 15-25%) in 2005. **Countries at a high risk of inadequate zinc intake (estimated prevalence >25%) in 2005.
